# Frequent contacts to Emergency Medical Services (EMS): more than frequent callers

**DOI:** 10.1186/s12873-024-01104-9

**Published:** 2024-10-12

**Authors:** Astrid K. V. Harring, Ola Graesli, Kristin Häikiö, Magnus Hjortdahl, Trine M. Jørgensen

**Affiliations:** 1 Department for prehospital education and research, Institute for Nursing and Health promotion, Oslo Metropolitan University, PB 4, St. Olavs plass, Oslo, 0130 Norway; 2 Division of Prehospital Services, Oslo University Hospital, Oslo, Norway

## Abstract

**Background:**

A frequent caller is defined by The Frequent Caller National Network (FreCaNN) as an adult who makes five or more emergency calls in a month or twelve or more in three months, related to individual episodes of care. However, we believe that when limiting the definition to those who call themselves, one underestimates the impact frequent contacts have on the Emergency Medical Communication Center (EMCC) and the Emergency Medical Services (EMS).

**Method:**

We conducted a descriptive retrospective cross-sectional database review of frequent contacts; defined as persons who have ≥ 5 contacts in a month or ≥ 12 contacts in three months. Data were provided from Oslo EMCC, between 1. January 2017 and 31. December 2022. Contrary to the FreCaNN definition, we included all types of contacts and callers, both emergent and non-emergent, regarding patients of all ages.

**Results:**

During the study period, 2.149.400 contacts were registered. Of these 129.700 were contacts from frequent callers, where the patients called themselves. When including contacts frequently made on behalf of a patient, we found that 268.723 fit the definition of frequent emergency contacts. When also taking non-emergent contacts into account, a total of 437.361 contacts fit the definition of frequent contacts.

**Conclusion:**

When limiting the criteria to only frequent callers, one underestimates the impact persons who have frequent contacts, have on the EMCC and the EMS. We were able to distinguish between three categories—contacts from frequent callers, frequent emergency contacts, and frequent contacts. We believe broadening the definition can benefit both research and audits, when accessing the use of emergency resources to patients with frequent requests for help to the EMCCs.

**Supplementary Information:**

The online version contains supplementary material available at 10.1186/s12873-024-01104-9.

## Introduction

Emergency Medical Services (EMS) are necessary to preserve the public health at large. They handle life-threatening or acute conditions and are accessible in most countries, for anyone, at any time, by calling the emergency number. In some countries, there is a joint emergency number for fire, police, and medical services, while others, like Norway, have separate numbers for each service, directing medical calls to the nearest emergency medical communication centres (EMCC).

However, some people call the emergency number quite regularly, and the term *chronic caller* first appeared in medical literature in the early 1970s [1]. The Frequent Caller National Network (FreCaNN) defines *frequent callers* as ‘An individual aged 18 or over who makes five or more emergency calls relating to individual episodes of care in a month or twelve or more emergency calls related to individual episodes of care in three months’ [2]. While this definition focuses on individual callers, we know from clinical experience that not all frequent callers call on behalf of themselves. A systematic review published by Scott et al. in 2014 [3], could not find any studies that focused on the characteristics of callers to the EMS. However, a more recent qualitative study by Evans et al. [4] described a variety of situations in which others frequently called on the patients’ behalf, supporting the clinical impression. Furthermore, Maruster et al. [5] found many frequent users of care services to be “network users”, relying on multiple regional care providers. In their study, they demonstrated the potential and benefit of utilising EMS data to assess and quantify the number of frequent users and their associated calls.

The aim of this study was to determine how broadening the current frequent caller definition to include all types of callers and types of contacts (emergency calls, transport requests and web-orders for ambulances) would affect the reported prevalence of frequent contacts to the EMCC, and how such a change would influence key statistics such as age, gender, and reason for contact.

## Method

We conducted a descriptive, retrospective, cross-sectional, database review of frequent contacts to Oslo EMCC between 1. January 2017 and 31. December 2022.

### Setting

Oslo EMCC is the largest EMCC in Norway, encompassing the capital and surrounding municipalities. It covers 31% of the Norwegian population [6] and receives approximately 25% (248.000) of the medical emergency calls annually [7]. This number of emergency calls does not include other kinds of contacts, such as transport requests and web-orders.

In Norway, 90% of the medical emergency calls must be answered within 10 s [8]. The calls go directly to an EMCC medical operator (registered nurse or paramedic) who triage the call, instruct in first aid, and decide what medical help is needed and to what urgency. This process is guided by “The Norwegian Index for Emergency Medical Assistance” [9], a criteria-based dispatch protocol. This Index is symptom based, divided into 39 criteria cards. The operator finds the appropriate criteria card and assesses the patient’s condition from the most acute to the least urgent criteria. EMCC operators have a relatively high self-reported use of this support tool [10]. Dispatch priorities are color-coded: red for acute or potentially life-threatening conditions (priority 1), yellow for urgent conditions (priority 2), and green for non-emergent conditions (priority 3). If an EMS response is required, an EMCC resource coordinator allocates and dispatches the necessary resource, including Helicopter Emergency Services (HEMS) or Search-and-Rescue (SAR) if needed. Low-acuity emergency calls (priority 2 and 3) can be transferred to the local out-of-hours clinic (OOHC) for further evaluation [11]. The EMCC and EMS also serve as an integral part of the Norwegian healthcare system, coordinating and conducting urgent and planned EMS and HEMS transports and transfers.

### Inclusion criteria

When the EMCC receives a medical emergency call, transport request, or web-order for an ambulance, a contact is registered in the in the Acute Medical Information System (AMIS). There can be several calls or callers for each contact (e.g. calling to check up on progress, providing additional information). The system also allows one contact to have more than one patient associated with the same incidence.

In this study, the patient’s identity associated with each contact, referred to as ‘patient ID’ (i.e., social security number) in AMIS, was used to identify frequent contacts. Frequent contacts were defined as patients with ≥ 5 contacts in a month or ≥ 12 contacts in three months. Patient IDs who met the criteria were exported from the database to a dataset (*see data handling*). Contacts with unknown, only partial, or foreign identification number were excluded. The medical operator states the type of caller in AMIS (*see variables*), and we used this categorisation to identify whether the patient called themselves or if someone called on their behalf. The dataset included all types of callers and contacts (including web-orders), both emergent and non-emergent. While the FreCaNN definition states individuals over the age of 18 years, the anonymised dataset allowed us to include all ages, including paediatric patients, as they are rarely studied [12].

### Variables

Year of birth was chosen in the data extraction, as it is consistent over the six-year study period, whereas age is not. In this paper, *age* is therefore determined by subtracting year of the contact from year of birth. The term *gender* is used throughout the study, as the dataset used the Norwegian population registry, as individuals can apply to legally change gender in Norway.

Callers were classified by the medical operators into categories based on their relation to the patient (e.g. the patient, next of kin, the public, neighbour, the police, fire department). *Healthcare personnel* include a variety of contacts such as phone or web-orders for planned or urgent transport/transfers, emergency calls from healthcare personnel in primary care such as homecare, nursing homes, assisted living or specialist care such as psychiatric centres and private hospitals without acute care. Whereas contacts from the OOHC can either be that the OOHC telephone nurse/operator wish to transfer the call to the EMCC medical operator, or request transport after the patient is seen by a doctor. Similarly, when the caller is a doctor, it is often a request for transport, mostly an admission.

### Categories of contacts

We divided the EMCC data into three categories by gradual inclusion:

Category 1: *Contacts from frequent callers*, the current FreCaNN definition, were contacts in which the patient calls the medical emergency number 1-1-3 themselves.

Category 2: *Frequent emergency contacts* were contacts made by all types of callers, to the medical emergency number 1-1-3.

Category 3: *Frequent contacts* were all types of callers and all types of contacts (emergency calls, transport requests and web-orders for ambulances), including non-emergent contacts.

### Data handling and analysis

The data provider, Oslo University Hospital, checked for duplicates, anonymised the dataset, assigned a study ID-number to each unique identity, and delivered the dataset to their secure research server, where all data handling has been executed. All analysis was done in SPSS Version 29.0.0.0 (IBM Corporation) and are presented as descriptive statistics, that is, frequencies, percentages, and age as median with interquartile range (IQR). Categorical variables were compared using Chi square test with a significance level set to a P-value of ≤ 0.05.

### Ethics

We submitted a pre-approval application to The Regional Medical Ethics committee, who considered the study to be regarded as quality assurance and improvement and thus outside of their scope, according to the Health Research Act (nr. 263844). The Norwegian directory of Health waived the requirement for consent by participants (nr. 23/7305-2). The Data Protection Official at Oslo University Hospital approved the study (nr. 21/14225).

## Results

**Fig. 1:**
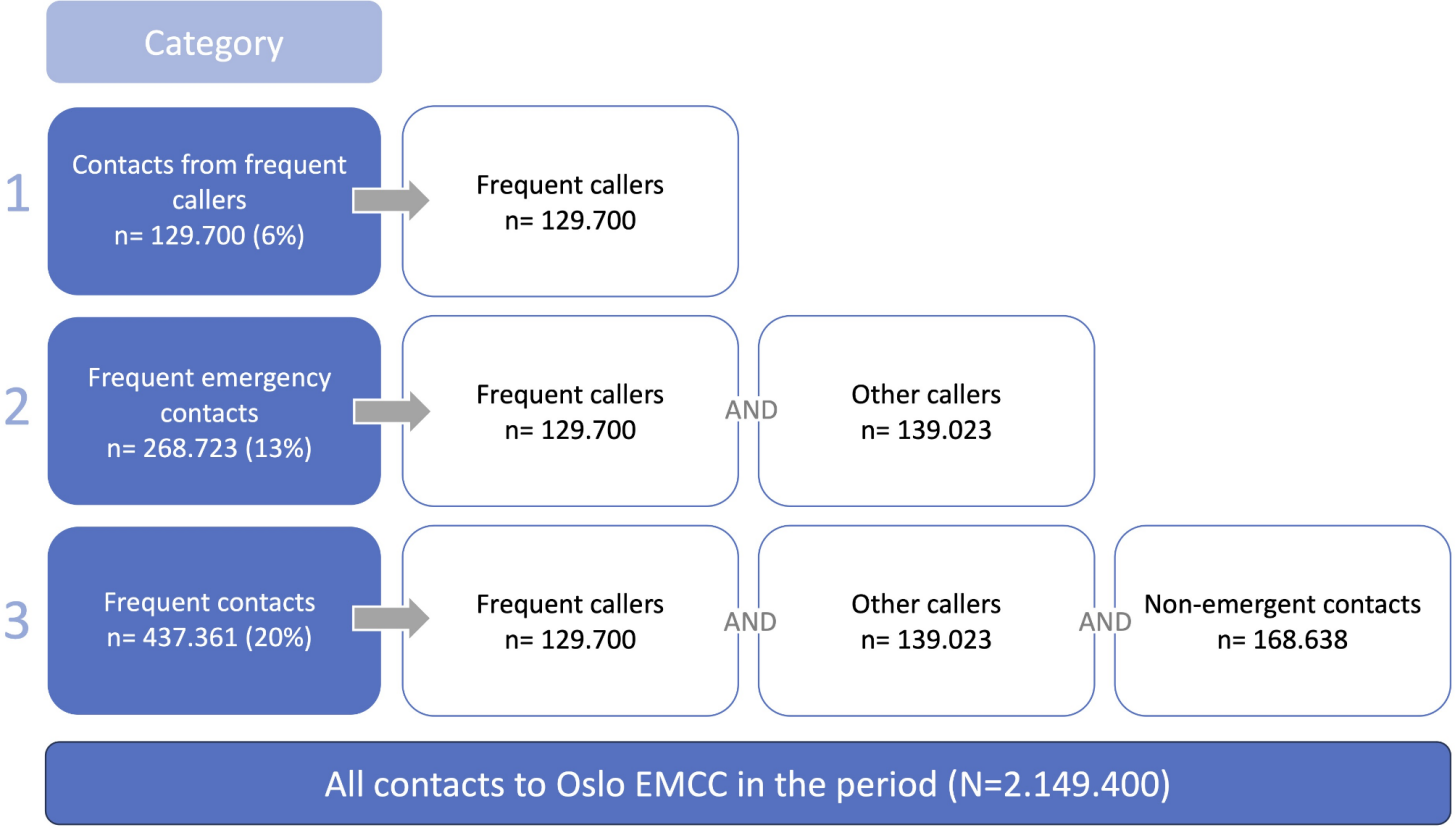
Number of contacts for the three categories of frequent contact (*N* = 2.149.400). The data can be divided into three categories: frequent contacts, frequent emergency contacts and contacts from frequent callers

In the six-year study period, Oslo EMCC registered 2.149.400 contacts (Fig. 1). In 129.700 (6%) instances, the frequent emergency contacts were initiated by the patients themselves. When considering all types of callers, we identified 268.723 (13%) frequent emergency contacts. Expanding the inclusion to non-emergency contacts, a total of 437.361 (20%) met the criteria for frequent contacts, finding that the EMCC was frequently contacted on the patient’s behalf by others, such as their next of kin or healthcare personnel (Table 1).

**Table 1:** Type of caller according to category

	Contacts from frequent callers		Frequent emergency contacts		Frequent contacts	
Number of contacts	*n*	%	*n*	%	*n*	%
Patient	129.700	100	129.700	48	135.073	31
Next of kin	-		46.299	17	49.077	11
Healthcare personnel, combined	-		54.043	21	181.327	45
Other healthcare personnel*	-		44.381	17	156.115	36
OOHC	-		5.881	2.2	11.457	2.6
Doctor	-		3.781	1.4	28.851	6.6
Others**	-		31.013	12	45.289	10
N/A	-		7.668	2.9	11.499	2.6
**Total number of contacts**	**129.700**	**100**	**268.723**	**100**	**437.361**	**100**

*Includes web-orders

** Includes e.g. the public, neighbours, the police, fire department. OOHC: out-of-hours clinic, N/A: not applicable or unknown

**Fig. 2:**
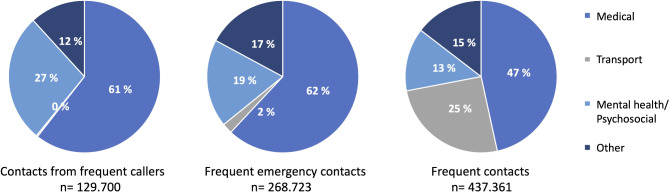
Reason for contact according to category. Other include i.e.: Accidents, major and minor injuries, intoxication, non-emergent medical issues, chief complaint not applicable

As seen in Fig. 2, the three categories had different relative distributions of reasons for contacting the EMCC. Whereas transport requests constituted 0.3% in contacts from frequent callers, they were 25% for frequent contacts. Medical emergencies and mental health- or psychosocial-related contacts showed the opposite pattern.

Ambulance use increased from 31% in contacts from frequent callers, to 53% in frequent emergency contacts, and to 67% in frequent contacts (see supplementary table). For category 2 and 3 there was a significant difference between type of caller and whether an ambulance had been dispatched (*p* < .001). There was also a significant difference between the reason for contact and the type of caller (*p* < .001). For instance, healthcare personnel combined are responsible for 108.862 (97%) of the total 111.849 contacts regarding transports.

**Fig. 3:**
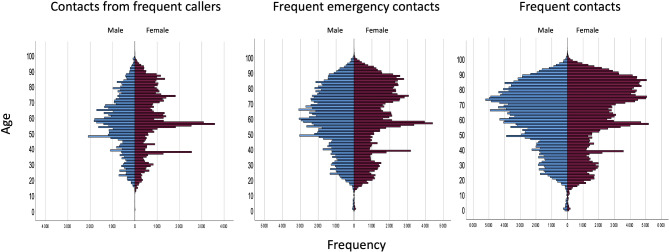
Age distribution according to gender and category

Figure 3 present the distribution of patients across all ages, including children. The median age of the patients increased between categories, from 59 (IQR: 26) to 62 (IQR: 31) to 66 (IQR: 31). In terms of patients’ gender, it ranged from 53% female in contacts from frequent callers to 50% female in frequent contacts.

## Discussion

By including all types of contacts and callers to the EMCC, we were able to describe three categories that have an impact on the use of EMCC and EMS resources: contacts from frequent callers, frequent emergency contacts, and frequent contacts. Our results demonstrate that healthcare personnel and others frequently contact the EMCC on the patient’s behalf.

The first category, which traditionally is defined as *frequent callers*, regards only those who repeatedly call for themselves. Even if such a definition seems specific, the group encompassed in this category is heterogenous [15], and most have multiple and complex needs, requiring various interventions, regarding both their medical and mental health condition, and unmet personal or social care needs [16]. One must be aware that this category and the current definition excludes patients with similar needs and resource demands, where others are concerned on their behalf.

The second category, *frequent emergency contacts*, includes calls directed to the medical emergency number (1–1–3). We found an increase in emergency calls made by patients’ next of kin, neighbours, or other members of the public. The volume of contacts in this category, supports our hypothesis that there are patients whom the current definition fails to address. Though they do not call themselves, they nevertheless constitute a high demand of resources at the EMCCs.

There are few descriptions of patients considered frequent callers who do not make the calls themselves, indicating a need for further elaboration and research. One such individual was recently described extensively in a report called “nobody can help me” by the Norwegian Healthcare Investigation Board [19], and several varieties of how and why others call on a patient’s behalf were found in a qualitative study of people who frequently call the emergency ambulance service in the UK [4].

Surprisingly, there was still a high proportion of healthcare personnel calling on patients’ behalf. This category excludes most requests for ambulance transport; however, if a transport or transfer is deemed an emergency (priority 1), healthcare personnel are instructed to call 1-1-3 to not delay dispatch. Furthermore, some requests for urgent (priority 2), and non-urgent (priority 3) transports will be present in this category, as not all will have made transport requests using the assigned transport-phone number or web-order system for planned transports and transfers. Thus, some contacts from healthcare personnel are expected. However, when adding the three types of healthcare professionals (calling while at work) into one category (other healthcare personnel, OOHC, and doctors), healthcare professionals constitute 21% (Table 1) of the emergency calls who could be considered frequent, whilst only 2% of the reason for contact was reported to be due to transport requests in this category (Fig. 2).

The third category, *frequent contacts*, is the most relevant when exploring ambulance use. Usually frequent or repeated ambulance use [13, 14] and frequent callers [15, 16] are studied and reported separately, whereas Scott et al. [3] introduced “frequent callers to and users of EMS”. In this paper, we have called this category “frequent contacts”. Among all frequent contacts to the EMCC, healthcare personnel were the most common. In most cases, this appeared to be patients requiring frequent ambulance transport to, from, or between healthcare services. Thus, healthcare personnel are the ones ordering these transports through EMCC contacts.

Repeated ambulance use is associated with chronic health problems and a high level of comorbidity [13]. Ambulance transport requests have a high impact on EMS resources and are expected to increase due to an aging population, combined with a centralization and specialization of services, often making longer transports necessary. Therefore, we believe there is a need to explore other solutions for some patient groups to reduce the rate of frequent non-urgent transports as measures to increase available ambulances for those in urgent or emergent need. The reduction of long-term and planned ambulance use can stem from initiating other means of transportation, such as wheelchair taxies, and in other cases, by considering alternative options for patients where receiving home treatment could be mutually beneficial such as home dialysis [17] or digital wound programs [18].

### Future perspectives

It is unlikely that emergency services can adequately meet the complex and multifaceted needs of those in frequent contact with the EMCC. As Maruster et al. [5] points out, EMS could potentially take on a network role as the ‘ferryman’, overseeing and identifying frequent patients. In that way, a unified – joint interaction plan could be implemented that would ensure cross-sectoral collaboration and coordination between healthcare services [19]. Such initiatives should include social services, as complex and unmet needs might be part of the explanation for continuously seeking contact with the emergency ambulance services [4].

Furthermore, for both frequent emergency contacts and contacts from frequent callers’ physical symptoms and medical issues constitute most contacts (< 60%, Fig. 2). Emerging initiatives such as digital follow-up and home monitoring [20] could be beneficial for many of these patients, as there are interventions to enhance both physical and mental health. By detecting deterioration early and implementing an individualised treatment plan to reduce or relive their symptoms and complaints, services could improve patients’ quality of life, reducing their need for urgent care and preventing emergent admissions.

### Strengths and limitations

The study is based on a sole source. Even though Oslo EMCC is by far the largest centre, it is only one out of 16 EMCCs in Norway. Note that in this study, we only report the number of contacts, not the number of individual patients. We identified 5208 frequent contacts regarding patients under the age of 18 years, suggesting a need to address this group separately.

## Conclusion

We found that many frequent contacts do not come from patients themselves, and our results demonstrate that frequent contacts to the EMCC can be divided into three categories: contacts from frequent callers, frequent emergency contacts, and frequent contacts.

In future research and audits, one must be aware that when limiting the inclusion criteria to the current frequent caller definition, one underestimates the impact frequent contacts have on the EMCC and the EMS.

## Electronic supplementary material

Below is the link to the electronic supplementary material.

Supplementary Material 1

## Data Availability

Data for this study available on Oslo University Hospital secure research server and are available through the corresponding author on reasonable request.

## Abbreviations

1-1-3: Norway’s Medical Emergency Number
AMIS: Acute Medical Information System
EMCC: Emergency Medical Communication Centre
EMS: Emergency Medical Services
FreCaNN: The Frequent Caller National Network
GP: General Practitioner
HEMS: Helicopter Emergency Services
IQR: Interquartile Range
N/A: Not Applicable
OOHC: Out-of-hours clinic
SAR: Search-and-Rescue
