# Supplementary material for: Frequent contacts to Emergency Medical Services (EMS): more than frequent callers
Source: BMC Emerg Med. 2024 Oct 12;24:190. doi: 10.1186/s12873-024-01104-9 (PMC11470579; doi:10.1186/s12873-024-01104-9)
Supplement: Supplementary file 1 — Supplementary Material 1 [file 12873_2024_1104_MOESM1_ESM.docx]

| **Category** | **Contacts from frequent callers (n=129.700)** | **Frequent emergency contact**  **(n=268.723)** | | | | **Frequent contact**  **(n=437.361)** | | | |
| --- | --- | --- | --- | --- | --- | --- | --- | --- | --- |
| **Type of caller** | **Patient** | **Patient** | **Next of kin** | **Healthcare personnel, combined*** | **Other**** | **Patient** | **Next of kin** | **Healthcare personnel, combined*** | **Other**** |
| **Ambulance dispatched, n** | 40.329 | 40.329 | 31.904 | 46.169 | 24.380 | 44.306 | 34.172 | 178.281 | 34.596 |
| **Ambulance not dispatched, n** | 89.371 | 89.371 | 14.395 | 7.874 | 14.301 | 90.767 | 14.905 | 18.142 | 22.192 |
| **Total, n** | **129.700** | **129.700** | **46.299** | **54.043** | **38.681** | **135.073** | **49.077** | **196.423** | **56.788** |

Supplementary table: Crosstabulation between type of caller and whether ambulance was dispatched, according to category

*Includes web-orders.

** Includes e.g. the public, neighbours, the police, fire department, not applicable or unknown. OOHC: out-of-hours clinic
